# Solving Mean-Payoff Games via Quasi Dominions

**DOI:** 10.1007/978-3-030-45237-7_18

**Published:** 2020-03-13

**Authors:** Massimo Benerecetti, Daniele Dell’Erba, Fabio Mogavero

**Affiliations:** 8 Johannes Kepler University, Linz, Austria; 9 University of Birmingham, Birmingham, UK; Università degli Studi di Napoli Federico II, Naples, Italy

## Abstract

We propose a novel algorithm for the solution of *mean-payoff games* that merges together two seemingly unrelated concepts introduced in the context of parity games, *small progress measures* and *quasi dominions*. We show that the integration of the two notions can be highly beneficial and significantly speeds up convergence to the problem solution. Experiments show that the resulting algorithm performs orders of magnitude better than the asymptotically-best solution algorithm currently known, without sacrificing on the worst-case complexity.

### 1 Introduction

In this article we consider the problem of solving *mean-payoff games*, namely infinite-duration perfect-information two-player games played on weighted directed graphs, each of whose vertexes is controlled by one of the two players. The game starts at an arbitrary vertex and, during its evolution, each player can take moves at the vertexes it controls, by choosing one of the outgoing edges. The moves selected by the two players induce an infinite sequence of vertices, called play. The payoff of any prefix of a play is the sum of the weights of its edges. A play is winning if it satisfies the game objective, called *mean-payoff objective*, which requires that the limit of the *mean payoff*, taken over the prefixes lengths, never falls below a given *threshold* $\nu$.

Mean-payoff games have been first introduced and studied by Ehrenfeucht and Mycielski in [20], who showed that positional strategies suffice to obtain the optimal value. A slightly generalized version was also considered by Gurvich *et al.* in [24]. Positional determinacy entails that the decision problem for these games lies in NPTime ∩ CoNPTime [34], and it was later shown to belong to UPTime ∩ CoUPTime [25], being UPTime the class of unambiguous nondeterministic polynomial time. This result gives the problem a rather peculiar complexity status, shared by very few other problems, such as integer factorization [22], [1] and parity games [25]. Despite various attempts [7, 19, 24, 30, 34], no polynomial-time algorithm for the mean-payoff game problems is known so far.

A different formulation of the game objective allows to define another class of quantitative games, known as *energy games*. The *energy objective* requires that, given an initial value $c$, called *credit*, the sum of $c$ and the *payoff* of every prefix

* Partially supported by GNCS 2019 & 2020 projects "Metodi Formali per Tecniche di Verifica Combinata" and "Ragionamento Strategico e Sintesi Automatica di Sistemi Multi-Agente".

of the play never falls below 0. These games, however, are tightly connected to mean-payoff games, as the two type of games have been proved to be log-space equivalent [11]. They are also related to other more complex forms of quantitative games. In particular, unambiguous polynomial-time reductions [25] exist from these games to *discounted payoff* [34] and *simple stochastic games* [18].

Recently, a fair amount of work in formal verification has been directed to consider, besides correctness properties of computational systems, also quantitative specifications, in order to express performance measures and resource requirements, such as quality of service, bandwidth and power consumption and, more generally, bounded resources. Mean-payoff and energy games also have important practical applications in system verification and synthesis. In [14] the authors show how quantitative aspects, interpreted as penalties and rewards associated to the system choices, allow for expressing optimality requirements encoded as mean-payoff objectives for the automatic synthesis of systems that also satisfy parity objectives. With similar application contexts in mind, [9] and [8] further contribute to that effort, by providing complexity results and practical solutions for the verification and automatic synthesis of reactive systems from quantitative specifications expressed in linear time temporal logic extended with mean-payoff and energy objectives. Further applications to temporal networks have been studied in [16] and [15]. Consequently, efficient algorithms to solve mean-payoff games become essential ingredients to tackle these problems in practice.

Several algorithms have been devised in the past for the solution of the decision problem for mean-payoff games, which asks whether there exists a strategy for one of the players that grants the mean-payoff objective. The very first deterministic algorithm was proposed in [34], where it is shown that the problem can be solved with $\mathrm{O}\big(n^3 \cdot m \cdot W\big)$ arithmetic operations, with $n$ and $m$ the number of positions and moves, respectively, and $W$ the maximal absolute weight in the game. A strategy improvement approach, based on iteratively adjusting a randomly chosen initial strategy for one player until a winning strategy is obtained, is presented in [31], which has an exponential upper bound. The algorithm by Lifshits and Pavlov [29], which runs in time $\mathrm{O}(n \cdot m \cdot 2^n \cdot \log_2 W)$, computes the "potential" of each game position, which corresponds to the initial credit that the player needs in order to win the game from that position. Algorithms based on the solution of linear feasibility problems over the tropical semiring have been also provided in [2–4]. The best known deterministic algorithm to date, which requires $\mathrm{O}(n \cdot m \cdot W)$ arithmetic operations, was proposed by Brim *et al.* [13]. They adapt to energy and mean-payoff games the notion of progress measures [28], as applied to parity games in [26]. The approach was further developed in [17] to obtain the same complexity bound for the optimal strategy synthesis problem. A strategy-improvement refinement of this technique has been introduced in [12]. Finally, Bjork *et al.* [6] proposed a randomized strategy-improvement based algorithm running in time $\mathsf{min}\{\mathrm{O}\big(n^2 \cdot m \cdot W\big), 2^{\mathrm{O}\left(\sqrt{n} \cdot \log n\right)}\}$.

Our contribution is a novel mean-payoff progress measure approach that enriches such measures with the notion of *quasi dominions*, originally introduced in [5] for parity games. These are sets of positions with the property that as long as the opponent chooses to play to remain in the set, it loses the game for sure, hence its best choice is always to try to escape. A quasi dominion from where escaping is not possible is a winning set for the other player. Progress measure approaches, such as the one of [13], typically focus on finding the best choices of the opponent and little information is gathered on the other player. In this sense, they are intrinsically asymmetric. Enriching the approach with quasi dominions can be viewed as a way to also encode the best choices of the player, information that can be exploited to speed up convergence significantly. The main difficulty here is that suitable lift operators in the new setting do not enjoy monotonicity. Such a property makes proving completeness of classic progress measure approaches almost straightforward, as monotonic operators do admit a least fixpoint. Instead, the lift operator we propose is only inflationary (specifically, non-decreasing) and, while still admitting fixpoints [10, 33], need not have a least one. Hence, providing a complete solution algorithm proves more challenging. The advantages, however, are significant. On the one hand, the new algorithm still enjoys the same worst-case complexity of the best known algorithm for the problem proposed in [13]. On the other hand, we show that there exist families of games on which the classic approach requires a number of operations that can be made arbitrarily larger than the one required by the new approach. Experimental results also witness the fact that this phenomenon is by no means isolated, as the new algorithm performs orders of magnitude better than the algorithm developed in [13].

### 2 Mean-Payoff Games

A two-player turn-based *arena* is a tuple $\mathcal{A} = \langle \mathrm{Ps}_\oplus, \mathrm{Ps}_\boxminus, Mv \rangle$, with $\mathrm{Ps}_\oplus \cap \mathrm{Ps}_\boxminus = \emptyset$ and $\mathrm{Ps} \triangleq \mathrm{Ps}_\oplus \cup \mathrm{Ps}_\boxminus$, such that $\langle \mathrm{Ps}, Mv \rangle$ is a finite directed graph without sinks. $\mathrm{Ps}_\oplus$ (*resp.*, $\mathrm{Ps}_\boxminus$) is the set of positions of player $\oplus$ (*resp.*, $\boxminus$) and $Mv \subseteq \mathrm{Ps} \times \mathrm{Ps}$ is a left-total relation describing all possible moves. A *path* in $\mathrm{V} \subseteq \mathrm{Ps}$ is a finite or infinite sequence $\pi \in \mathrm{Pth}(\mathrm{V})$ of positions in V compatible with the move relation, *i.e.*, $(\pi_i, \pi_{i+1}) \in Mv$, for all $i \in [0, |\pi| - 1)$. A positional *strategy* for player $\alpha \in \{\oplus, \boxminus\}$ on $\mathrm{V} \subseteq \mathrm{Ps}$ is a function $\sigma_\alpha \in \mathrm{Str}_\alpha(\mathrm{V}) \subseteq (\mathrm{V} \cap \mathrm{Ps}_\alpha) \to \mathrm{Ps}$, mapping each $\alpha$-position $v$ in the domain of $\sigma_\alpha$ to position $\sigma_\alpha(v)$ compatible with the move relation, *i.e.*, $(v, \sigma_\alpha(v)) \in Mv$. With $\mathrm{Str}_\alpha(\mathrm{V})$ we denote the set of all $\alpha$-strategies on V, while $\mathrm{Str}_\alpha$ denotes $\bigcup_{\mathrm{V} \subseteq \mathrm{Ps}} \mathrm{Str}_\alpha(\mathrm{V})$. A *play* in $\mathrm{V} \subseteq \mathrm{Ps}$ from a position $v \in \mathrm{V}$ *w.r.t.* a pair of strategies $(\sigma_\oplus, \sigma_\boxminus) \in \mathrm{Str}_\oplus(\mathrm{V}) \times \mathrm{Str}_\boxminus(\mathrm{V})$, called $((\sigma_\oplus, \sigma_\boxminus), v)$*-play*, is a path $\pi \in \mathrm{Pth}(\mathrm{V})$ such that $\pi_0 = v$ and, for all $i \in [0, |\pi| - 1)$, if $\pi_i \in \mathrm{Ps}_\oplus$ then $\pi_{i+1} = \sigma_\oplus(\pi_i)$ else $\pi_{i+1} = \sigma_\boxminus(\pi_i)$. The *play function* $\mathsf{play} : (\mathrm{Str}_\oplus(\mathrm{V}) \times \mathrm{Str}_\boxminus(\mathrm{V})) \times \mathrm{V} \to \mathrm{Pth}(\mathrm{V})$ returns, for each position $v \in \mathrm{V}$ and pair of strategies $(\sigma_\oplus, \sigma_\boxminus) \in \mathrm{Str}_\oplus(\mathrm{V}) \times \mathrm{Str}_\boxminus(\mathrm{V})$, the maximal $((\sigma_\oplus, \sigma_\boxminus), v)$-play $\mathsf{play}((\sigma_\oplus, \sigma_\boxminus), v)$. If a pair $(\sigma_\oplus, \sigma_\boxminus) \in \mathrm{Str}_\oplus(\mathrm{V}) \times \mathrm{Str}_\boxminus(\mathrm{V})$ induces a finite play starting from position $v \in \mathrm{V}$, then $\mathsf{play}((\sigma_\oplus, \sigma_\boxminus), v)$ identifies the maximal prefix of that play that is contained in V.

A *mean-payoff game* (MPG for short) is a tuple $\partial = \langle \mathcal{A}, \mathrm{Wg}, \mathsf{wg} \rangle$, where $\mathcal{A}$ is an arena, $\mathrm{Wg} \subset \mathbb{Z}$ is a finite set of integer weights, and $\mathsf{wg} \colon \mathrm{Ps} \to \mathrm{Wg}$ is a

*weight function* assigning a weight to each position. $\mathrm{Ps}^{+}$ (*resp.*, $\mathrm{Ps}^{-}$) denotes the set of positive-weight positions (*resp.*, non-positive-weight positions). For convenience, we shall refer to non-positive weights as negative weights. Notice that this definition of MPG is equivalent to the classic formulation in which the weights label the moves, instead. The weight function naturally extends to paths, by setting $\mathsf{wg}(\pi) \triangleq \sum_{i=0}^{|\pi|-1} \mathsf{wg}(\pi_i)$. The goal of player $\oplus$ (*resp.*, $\boxminus$) is to maximize (*resp.*, minimize) $\mathsf{v}(\pi) \triangleq \liminf_{i\to\infty} \frac{1}{i} \cdot \mathsf{wg}(\pi_{\leq i})$, where $\pi_{\leq i}$ is the prefix up to index $i$. Given a threshold $\nu$, a set of positions $\mathrm{V} \subseteq \mathrm{Ps}$ is a $\oplus$-*dominion*, if there exists a $\oplus$-strategy $\sigma_{\oplus} \in \mathrm{Str}_{\oplus}(\mathrm{V})$ such that, for all $\boxminus$-strategies $\sigma_{\boxminus} \in \mathrm{Str}_{\boxminus}(\mathrm{V})$ and positions $v \in \mathrm{V}$, the induced play $\pi = \mathsf{play}((\sigma_{\oplus}, \sigma_{\boxminus}), v)$ satisfies $\mathsf{v}(\pi) > \nu$. The pair of winning regions $(\mathrm{Wn}_{\oplus}, \mathrm{Wn}_{\boxminus})$ forms a $\nu$-mean partition. Assuming $\nu$ integer, the $\nu$-mean partition problem is equivalent to the 0-mean partition one, as we can subtract $\nu$ to the weights of all the positions. As a consequence, the MPG decision problem can be equivalently restated as deciding whether player $\oplus$ (*resp.*, $\boxminus$) has a strategy to enforce $\liminf_{i\to\infty} \frac{1}{i} \cdot \mathsf{wg}(\pi_{\leq i}) > 0$ (*resp.*, $\liminf_{i\to\infty} \frac{1}{i} \cdot \mathsf{wg}(\pi_{\leq i}) \leq 0$), for all the resulting plays $\pi$.

### 3 Solving Mean-Payoff Games via Progress Measures

The abstract notion of progress measure [28] has been introduced as a way to encode global properties on paths of a graph by means of simpler local properties of adjacent vertexes. In the context of MPGs, the graph property of interest, called *mean-payoff property*, requires that the mean payoff of every infinite path in the graph be non-positive. More precisely, in game theoretic terms, a *mean-payoff progress measure* witnesses the existence of strategy $\sigma_{\boxminus}$ for player $\boxminus$ such that each path in the graph induced by fixing that strategy on the arena satisfies the desired property. A mean-payoff progress measure associates with each vertex of the underlying graph a value, called *measure*, taken from the set of extended natural numbers $\mathbb{N}_{\infty} \triangleq \mathbb{N} \cup \{\infty\}$, endowed with an ordering relation $\leq$ and an addition operation $+$, which extend the standard ordering and addition over the naturals in the usual way. Measures are associated with positions in the game and the measure of a position $v$ can intuitively be interpreted as an estimate of the payoff that player $\oplus$ can enforce on the plays starting in $v$. In this sense, they measure "how far" $v$ is from satisfying the mean-payoff property, with the maximal measure $\infty$ denoting failure of the property for $v$. More precisely, the $\boxminus$-strategy induced by a progress measure ensures that measures do not increase along the paths of the induced graph. This ensures that every path eventually gets trapped in a non-positive-weight cycle, witnessing a win for player $\boxminus$.

To obtain a progress measure, one starts from some suitable association of position of the game with measures. The local information encoded by these measures is then propagated back along the edges of the underlying graph so as to associate with each position the information gathered along plays of some finite length starting from that position. The propagation process is performed according to the following intuition. The measures of positions adjacent to $v$ are propagated back to $v$ only if those measures push $v$ further away from the property. This propagation is achieved by means of a measure stretch operation $+$, which adds, when appropriate, the measure of an adjacent position to the weight of a given position. This is established by comparing the measure of $v$ with those of its adjacent positions, since, for each position $v$, the mean-payoff property is defined in terms of the sum of the weights encountered along the plays from that position. The process ends when no position can be pushed further away from the property and each position is not dominated by any, respectively one, of its adjacents, depending on whether that position belongs to player $\oplus$ or to player $\boxminus$, respectively. The positions that did not reach measure $\infty$ are those from which player $\boxminus$ can win the game and the set of measures currently associated with such positions forms a mean-payoff progress measure.

To make the above intuitions precise, we introduce the notion of measure function, progress measure, and an algorithm for computing progress measures correctly. It is worth noticing that the progress-measure based approach as described in [13], called SEPM from now on, can be easily recast equivalently in the form below. A *measure function* $\mu\colon \mathrm{Ps} \to \mathbb{N}_\infty$ maps each position $v$ in the game to a suitable measure $\mu(v)$. The order $\leq$ of the measures naturally induces a pointwise partial order $\sqsubseteq$ on the measure functions defined in the usual way, namely, for any two measure functions $\mu_1$ and $\mu_2$, we write $\eta_1 \sqsubseteq \eta_2$ if $\mu_1(v) \leq \mu_2(v)$, for all positions $v$. The set of measure functions over a measure space, together with the induced ordering $\sqsubseteq$, forms a *measure-function space.*

**Definition 1 (Measure-Function Space).** *The* measure-function space *is the* partial order $\mathcal{F} \triangleq \langle \mathrm{MF}, \sqsubseteq \rangle$ *whose components are defined as follows:*

1. $\mathrm{MF} \triangleq \mathrm{Ps} \to \mathbb{N}_\infty$ *is the set of all functions* $\mu \in \mathrm{MF}$*, called* measure functions*, mapping each position* $v \in \mathrm{Ps}$ *to a measure* $\mu(v) \in \mathbb{N}_\infty$*;*
2. *for all* $\mu_1, \mu_2 \in \mathrm{MF}$*, it holds that* $\mu_1 \sqsubseteq \mu_2$ *if* $\mu_1(v) \leq \mu_2(v)$*, for all* $v \in \mathrm{Ps}$.

*The* $\oplus$-denotation *(*resp.*,* $\boxminus$-denotation*) of a measure function* $\mu \in \mathrm{MF}$ *is the set* $\|\mu\|_\oplus \triangleq \mu^{-1}(\infty)$ *(*resp.*,* $\|\mu\|_\boxminus \triangleq \overline{\mu^{-1}(\infty)}$*) of all positions having maximal (*resp.*, non-maximal) measure associated within* $\mu$*.*

Consider a position $v$ with an adjacent $u$ with measure $\eta$. A measure update of $\eta$ *w.r.t.* $v$ is obtained by the stretch operator $+\colon \mathbb{N}_\infty \times \mathrm{Ps} \to \mathbb{N}_\infty$, defined as $\eta + v \triangleq \mathsf{max}\{0, \eta + \mathsf{wg}(v)\}$, which corresponds to the payoff estimate that the given position will obtain by choosing to follow the move leading to the $u$.

A *mean-payoff progress measure* is such that the measure associated with each game position $v$ need not be increased further in order to beat the actual payoff of the plays starting from $v$. In particular, it can be defined by taking into account the opposite attitude of the two players in the game. While the player $\oplus$ tries to push toward higher measures, the player $\boxminus$ will try to keep the measures as low as possible. A measure function in which the payoff of each $\oplus$-position (*resp.*, $\boxminus$-position) $v$ is not dominated by the payoff of all (*resp.*, some of) its adjacents augmented with the weight of $v$ itself meets the requirements.

**Definition 2 (Progress Measure).** *A measure function* $\mu \in \mathrm{MF}$ *is a* progress measure *if the following two conditions hold true, for all positions* $v \in \mathrm{Ps}$*:*

1. $\mu(u) + v \leq \mu(v)$, *for all adjacents* $u \in Mv(v)$ *of* $v$, *if* $v \in \mathrm{Ps}_{\oplus}$;
2. $\mu(u) + v \leq \mu(v)$, *for some adjacent* $u \in Mv(v)$ *of* $v$, *if* $v \in \mathrm{Ps}_{\boxminus}$.

The following theorem states the fundamental property of progress measures, namely, that every position with a non-maximal measures is won by player $\boxminus$.

**Theorem 1 (Progress Measure).** $\|\mu\|_{\boxminus} \subseteq \mathrm{Wn}_{\boxminus}$, *for all progress measures* $\mu$.

In order to obtain a progress measure from a given measure function, one can iteratively adjust the current measure values in such a way to force the progress condition above among adjacent positions. To this end, we define the *lift operator* $\mathsf{lift}\colon \mathrm{MF} \to \mathrm{MF}$ as follows:

$$\mathsf{lift}(\mu)(v) \triangleq \begin{cases} \max\{\mu(w) + v : w \in Mv(v)\}, & \text{if } v \in \mathrm{Ps}_{\oplus}; \\ \min\{\mu(w) + v : w \in Mv(v)\}, & \text{otherwise.} \end{cases}$$

Note that the lift operator is clearly monotone and, therefore, admits a least fixpoint. A mean-payoff progress measure can be obtained by repeatedly applying this operator until a fixpoint is reached, starting from the minimal measure function $\mu_0 \triangleq \{v \in \mathrm{Ps} \mapsto 0\}$ that assigns measure 0 to all the positions in the game. The following *solver operator* applied to $\mu_0$ computes the desired solution: $\mathsf{sol} \triangleq \mathsf{lfp}\,\mu\,.\,\mathsf{lift}(\mu)\colon \mathrm{MF} \to \mathrm{MF}$. Observe that the measures generated by the procedure outlined above have a fairly natural interpretation. Each positive measure, indeed, under-approximates the weight that player $\oplus$ can enforce along finite prefixes of the plays from the corresponding positions. This follows from the fact that, while player $\oplus$ maximizes its measures along the outgoing moves, player $\boxminus$ minimizes them. In this sense, each positive measure witnesses the existence of a positively-weighted finite prefix of a play that player $\oplus$ can enforce. Let $S \triangleq \sum\{\mathsf{wg}(v) \in \mathbb{N} : v \in \mathrm{Ps} \wedge \mathsf{wg}(v) > 0\}$ be the sum of all the positive weights in the game. Clearly, the maximal payoff of a simple play in the underlying graph cannot exceed $S$. Therefore, a measure greater than $S$ witnesses the existence of a cycle whose payoff diverges to infinity and is won, thus, by player $\oplus$. Hence, any measure strictly greater than $S$ can be substituted with the value $\infty$. This observation establishes the termination of the algorithm and is instrumental to its completeness proof. Indeed, at the fixpoint, the measures actually coincide with the highest payoff player $\oplus$ is able to guarantee. Soundness and completeness of the above procedure have been established in [13], where the authors also show that, despite the algorithm requiring $\mathrm{O}(n \cdot S) = \mathrm{O}\big(n^2 \cdot W\big)$ lift operations in the worst-case, with $n$ the number of positions and $W$ the maximal positive weight in the game, the overall cost of these lift operations is $\mathrm{O}(S \cdot m \cdot \log S) = \mathrm{O}(n \cdot m \cdot W \cdot \log(n \cdot W))$, with $m$ the number of moves and $\mathrm{O}(\log S)$ the cost of arithmetic operations to compute the stretch of the measures.

### 4 Solving Mean-Payoff Games via Quasi Dominions

Let us consider the simple example game depicted in Figure 1, where the shape of each position indicates the owner, circles for player $\oplus$ and square for its opponent $\boxminus$, and, in each label of the form $\ell/w$, the letter $w$ corresponds to the associated weight, where we assume $k > 1$. Starting from the smallest measure function $\mu_0 = \{\mathtt{a}, \mathtt{b}, \mathtt{c}, \mathtt{d} \mapsto 0\}$, the first application of the lift operator returns $\mu_1 = \{\mathtt{a} \mapsto k; \mathtt{b}, \mathtt{c} \mapsto 0; \mathtt{d} \mapsto 1\} = \mathsf{lift}(\mu_0)$. After that step, the following iterations of the fixpoint alternatively updates positions $\mathtt{c}$ and $\mathtt{d}$, since the other ones already satisfy the progress condition. Being $\mathtt{c} \in \mathrm{Ps}_{\boxminus}$, the lift operator chooses for it the measure computed along the move $(\mathtt{c}, \mathtt{d})$, thus obtaining $\mu_2(\mathtt{c}) = \mathsf{lift}(\mu_1)(\mathtt{c}) = \mu_1(\mathtt{d}) = 1$. Subsequently, $\mathtt{d}$ is updated to $\mu_3(\mathtt{d}) = \mathsf{lift}(\mu_2)(\mathtt{d}) = \mu_2(\mathtt{c}) + 1 = 2$. A progress measure is obtained after exactly $2k+1$ iterations, when the measure of $\mathtt{c}$ reaches value $k$ and $\mathtt{d}$ value $k+1$. Note, however, that the choice of the move $(\mathtt{c}, \mathtt{d})$ is clearly a losing strategy for player $\boxminus$, as remaining in the highlighted region would make the payoff from position $\mathtt{c}$ diverge. Therefore, the only reasonable choice for player $\boxminus$ is to exit from that region by taking the move leading to position $\mathtt{a}$. An operator able to diagnose this phenomenon early on could immediately discard the move $(\mathtt{c}, \mathtt{d})$ and jump directly to the correct payoff obtained by choosing the move to position $\mathtt{a}$. As we shall see, such an operator might lose the monotonicity property and recovering the completeness of the resulting approach will prove more involved.

In the rest of this article we devise a progress operator that does precisely that. We start by providing a notion of *quasi dominion*, originally introduced for parity games in [5], which can be exploited in the context of MPGs.

**Definition 3 (Quasi Dominion).** *An set of positions* $\mathrm{Q} \subseteq \mathrm{Ps}$ *is a* quasi $\oplus$-dominion *if there exists a* $\oplus$*-strategy* $\sigma_{\oplus} \in \mathrm{Str}_{\oplus}(\mathrm{Q})$*, called* $\oplus$-witness for $\mathrm{Q}$*, such that, for all* $\boxminus$*-strategies* $\sigma_{\boxminus} \in \mathrm{Str}_{\boxminus}(\mathrm{Q})$ *and positions* $v \in \mathrm{Q}$*, the play* $\pi = \mathsf{play}((\sigma_{\oplus}, \sigma_{\boxminus}), v)$*, called* $(\sigma_{\oplus}, v)$-play in $\mathrm{Q}$*, satisfies* $\mathsf{wg}(\pi) > 0$*. If the condition* $\mathsf{wg}(\pi) > 0$ *holds only for infinite plays* $\pi$*, then* $\mathrm{Q}$ *is called* weak quasi $\oplus$-dominion*.*

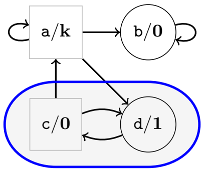

Fig. 1: An MPG.

Essentially, a quasi $\oplus$-dominion consists in a set $\mathrm{Q}$ of positions starting from which player $\oplus$ can force plays in $\mathrm{Q}$ of positive weight. Analogously, any infinite play that player $\oplus$ can force in a weak quasi $\oplus$-dominion has positive weight. Clearly, any quasi $\oplus$-dominion is also a weak quasi $\oplus$-dominion. Moreover, the latter are closed under subsets, while the former are not. It is an immediate consequence of the definition above that all infinite plays induced by the $\oplus$-witness, if any, necessarily have infinite weight and, thus, are winning for player $\oplus$. Indeed, every such a play $\pi$ is regular, *i.e.* it can be decomposed into a prefix $\pi'$ and a simple cycle $(\pi'')^{\omega}$, *i.e.* $\pi = \pi'(\pi'')^{\omega}$, since the strategies we are considering are memoryless. Now, $\mathsf{wg}((\pi'')^{\omega}) > 0$, so, $\mathsf{wg}(\pi'') > 0$, which implies $\mathsf{wg}((\pi'')^{\omega}) = \infty$. Hence, $\mathsf{wg}(\pi) = \infty$.

**Proposition 1.** *Let* $\mathrm{Q}$ *be a weak quasi* $\oplus$*-dominion with* $\sigma_{\oplus} \in \mathrm{Str}_{\oplus}(\mathrm{Q})$ *one of its* $\oplus$*-witnesses and* $\mathrm{Q}^{\star} \subseteq \mathrm{Q}$*. Then, for all* $\boxminus$*-strategies* $\sigma_{\boxminus} \in \mathrm{Str}_{\boxminus}(\mathrm{Q}^{\star})$ *and positions* $v \in \mathrm{Q}^{\star}$ *the following holds: if the* $(\sigma_{\oplus \upharpoonright \mathrm{Q}^{\star}}, v)$*-play* $\pi = \mathsf{play}((\sigma_{\oplus \upharpoonright \mathrm{Q}^{\star}}, \sigma_{\boxminus}), v)$ *is infinite, then* $\mathsf{wg}(\pi) = \infty$*.*

From Proposition 1, it directly follows that, if a weak quasi $\oplus$-dominion Q is *closed w.r.t.* its *$\oplus$-witness*, namely all the induced plays are infinite, then it is a $\oplus$-dominion, hence is contained in $\mathrm{Wn}_{\oplus}$.

Consider again the example of Figure 1. The set of position $\mathrm{Q} \triangleq \{\mathtt{a}, \mathtt{c}, \mathtt{d}\}$ forms a quasi $\oplus$-dominion whose $\oplus$-witness is the only possible $\oplus$-strategy mapping position $\mathtt{d}$ to $\mathtt{c}$. Indeed, any infinite play remaining in Q forever and compatible with that strategy (*e.g.*, the play from position $\mathtt{c}$ when player $\boxminus$ chooses the move from $\mathtt{c}$ leading to $\mathtt{d}$ or the one from $\mathtt{a}$ to itself or the one from $\mathtt{a}$ to $\mathtt{d}$) grants an infinite payoff. Any finite compatible play, instead, ends in position $\mathtt{a}$ (*e.g.*, the play from $\mathtt{c}$ when player $\boxminus$ chooses the move from $\mathtt{c}$ to $\mathtt{a}$ and then one from $\mathtt{a}$ to $\mathtt{b}$) giving a payoff of at least $k > 0$. On the other hand, $\mathrm{Q}^{\star} \triangleq \{\mathtt{c}, \mathtt{d}\}$ is only a weak quasi $\oplus$-dominion, as player $\boxminus$ can force a play of weight 0 from position $\mathtt{c}$, by choosing the exiting move $(\mathtt{c}, \mathtt{a})$. However, the internal move $(\mathtt{c}, \mathtt{d})$ would lead to an infinite play in $\mathrm{Q}^{\star}$ of infinite weight.

The crucial observation here is that the best choice for player $\boxminus$ in any position of a (weak) quasi $\oplus$-dominion is to exit from it as soon as it can, while the best choice for player $\oplus$ is to remain inside it as long as possible. The idea of the algorithm we propose in this section is to precisely exploit the information provided by the quasi dominions in the following way. Consider the example above. In position $\mathtt{a}$ player $\boxminus$ must choose to exit from $\mathrm{Q} = \{\mathtt{a}, \mathtt{c}, \mathtt{d}\}$, by taking the move $(\mathtt{a}, \mathtt{b})$, without changing its measure, which would corresponds to its weight $k$. On the other hand, the best choice for player $\boxminus$ in position $\mathtt{c}$ is to exit from the weak quasi-dominion $\mathrm{Q}^{\star} = \{\mathtt{c}, \mathtt{d}\}$, by choosing the move $(\mathtt{c}, \mathtt{a})$ and lifting its measure from 0 to $k$. Note that this contrasts with the minimal measure-increase policy for player $\boxminus$ employed in [13], which would keep choosing to leave $\mathtt{c}$ in the quasi-dominion by following the move to $\mathtt{d}$, which gives the minimal increase in measure of value 1. Once $\mathtt{c}$ is out of the quasi-dominion, though, the only possible move for player $\oplus$ is to follow $\mathtt{c}$, taking measure $k + 1$. The resulting measure function is the desired progress measure.

In order to make this intuitive idea precise, we need to be able to identify quasi dominions first. Interestingly enough, the measure functions $\mu$ defined in the previous section do allow to identify a quasi dominion, namely the set of positions $\overline{\mu^{-1}(0)}$ having positive measure. Indeed, as observed at the end of that section, a positive measure witnesses the existence of a positively-weighted finite play that player $\oplus$ can enforce from that position onward, which is precisely the requirement of Definition 3. In the example of Figure 1, $\overline{\mu_0^{-1}(0)} = \emptyset$ and $\overline{\mu_1^{-1}(0)} = \{\mathtt{a}, \mathtt{c}, \mathtt{d}\}$ are both quasi dominions, the first one *w.r.t.* the empty $\oplus$-witness and the second one *w.r.t.* the $\oplus$-witness $\sigma_{\oplus}(\mathtt{d}) = \mathtt{c}$.

We shall keep the quasi-dominion information in pairs $(\mu, \sigma)$, called *quasi-dominion representations* (QDR, for short), composed of a measure function $\mu$ and a $\oplus$-strategy $\sigma$, which corresponds to one of the $\oplus$-witnesses of the set of positions with positive measure in $\mu$. The connection between these two components is formalized in the definition below that also provides the partial order over which the new algorithm operates.

**Definition 4 (QDR Space).** *The* quasi-dominion-representation space *is the* partial order $\mathcal{Q} \triangleq \langle \mathrm{QDR}, \sqsubseteq \rangle$*, whose components are defined as follows:*

1. $\mathrm{QDR} \subseteq \mathrm{MF} \times \mathrm{Str}_{\oplus}$ *is the set of all pairs* $\varrho \triangleq (\mu_{\varrho}, \sigma_{\varrho}) \in \mathrm{QDR}$*, called* quasi-dominion-representations*, composed of a measure function* $\mu_{\varrho} \in \mathrm{MF}$ *and a* $\oplus$*-strategy* $\sigma_{\varrho} \in \mathrm{Str}_{\oplus}(\mathsf{Q}(\varrho))$*, where* $\mathsf{Q}(\varrho) \triangleq \overline{\mu_{\varrho}^{-1}(0)}$*, for which:*
   *(a)* $\mathsf{Q}(\varrho)$ *is a quasi* $\oplus$*-dominion enjoying* $\sigma_{\varrho}$ *as a* $\oplus$*-witness;*
   *(b)* $\|\mu_{\varrho}\|_{\oplus}$ *is a* $\oplus$*-dominion;*
   *(c)* $\mu_{\varrho}(v) \leq \mu_{\varrho}(\sigma_{\varrho}(v)) + v$*, for all* $\oplus$*-positions* $v \in \mathsf{Q}(\varrho) \cap \mathrm{Ps}_{\oplus}$*;*
   *(d)* $\mu_{\varrho}(v) \leq \mu_{\varrho}(u) + v$*, for all* $\boxminus$*-positions* $v \in \mathsf{Q}(\varrho) \cap \mathrm{Ps}_{\boxminus}$ *and* $u \in Mv(v)$*;*
2. *for all* $\varrho_1, \varrho_2 \in \mathrm{QDR}$*, it holds that* $\varrho_1 \sqsubseteq \varrho_2$ *if* $\mu_{\varrho_1} \sqsubseteq \mu_{\varrho_2}$ *and* $\sigma_{\varrho_1}(v) = \sigma_{\varrho_2}(v)$*, for all* $\oplus$*-positions* $v \in \mathsf{Q}(\varrho_1) \cap \mathrm{Ps}_{\oplus}$ *with* $\mu_{\varrho_1}(v) = \mu_{\varrho_2}(v)$*.*

*The* $\alpha$-denotation $\|\varrho\|_{\alpha}$ *of a* QDR $\varrho$*, with* $\alpha \in \{\oplus, \boxminus\}$*, is the* $\alpha$*-denotation* $\|\mu_{\varrho}\|_{\alpha}$ *of its measure function.*

Condition 1a is obvious. Condition 1b, instead, requires that every position with infinite measure is indeed won by player $\oplus$ and is crucial to guarantee the completeness of the algorithm. Finally, Conditions 1c and 1d ensure that every positive measure under approximates the actual weight of some finite play within the induced quasi dominion. This is formally captured by the following proposition, which can be easily proved by induction on the length of the play.

**Proposition 2.** *Let* $\varrho$ *be a* QDR *and* $v\pi u$ *a finite path starting at position* $v \in \mathrm{Ps}$ *and terminating in position* $u \in \mathrm{Ps}$ *compatible with the* $\oplus$*-strategy* $\sigma_{\varrho}$*. Then,* $\mu_{\varrho}(v) \leq \mathsf{wg}(v\pi) + \mu_{\varrho}(u)$*.*

It is immediate to see that every MPG admits a non-trivial QDR space, since the pair $(\mu_0, \sigma_0)$, with $\mu_0$ the smallest measure function and $\sigma_0$ the empty strategy, trivially satisfies all the required conditions.

**Proposition 3.** *Every* MPG *has a non-empty* QDR *space associated with it.*

The solution procedure we propose, called QDPM from now on, can intuitively be broken down as an alternation of two phases. The first one tries to lift the measures of positions outside the quasi dominion $\mathsf{Q}(\varrho)$ in order to extend it, while the second one lifts the positions inside $\mathsf{Q}(\varrho)$ that can be forced to exit from it by player $\boxminus$. The algorithm terminates when no new position can be absorbed within the quasi dominion and no measure needs to be lifted to allow the $\boxminus$-winning positions to exit from it, when possible. To this end, we define a controlled lift operator $\mathsf{lift} \colon \mathrm{QDR} \times 2^{\mathrm{Ps}} \times 2^{\mathrm{Ps}} \rightharpoonup \mathrm{QDR}$ that works on QDRs and takes two additional parameters, a source and a target set of positions. The intended meaning is that we want to restrict the application of the lift operation to the positions in the source set S, while using only the moves leading to the target set T. The different nature of the two types of lifting operations is reflected in the actual values of the source and target parameters.

$$\mathsf{lift}(\varrho, \mathrm{S}, \mathrm{T}) \triangleq \varrho^{\star}, \text{ where}$$

$$\mu_{\varrho^\star}(v) \triangleq \begin{cases} \mathsf{max}\{\mu_\varrho(u) + v : u \in Mv(v) \cap \mathrm{T}\}, & \text{if } v \in \mathrm{S} \cap \mathrm{Ps}_\oplus; \\ \mathsf{min}\{\mu_\varrho(u) + v : u \in Mv(v) \cap \mathrm{T}\}, & \text{if } v \in \mathrm{S} \cap \mathrm{Ps}_\boxminus; \\ \mu_\varrho(v), & \text{otherwise}; \end{cases}$$

and, for all $\oplus$-positions $v \in \mathsf{Q}(\varrho^\star) \cap \mathrm{Ps}_\oplus$, we choose $\sigma_{\varrho^\star}(v) \in \mathsf{argmax}_{u \in Mv(v) \cap \mathrm{T}}$ $\mu_\varrho(u) + v$, if $\mu_{\varrho^\star}(v) \neq \mu_\varrho(v)$, and $\sigma_{\varrho^\star}(v) = \sigma_\varrho(v)$, otherwise. Except for the restriction on the outgoing moves considered, which are those leading to the targets in T, the lift operator acts on the measure component of a QDR very much like the original lift operator does. In order to ensure that the result is still a QDR, however, the lift operator must also update the $\oplus$-witness of the quasi dominion. This is required to guarantee that Conditions 1a and 1c of Definition 4 are preserved. If the measure of a $\oplus$-position $v$ is not affected by the lift, the $\oplus$-witness must not change for that position. However, if the application of the lift operation increases the measure, then the $\oplus$-witness on $v$ needs to be updated to any move $(v, u)$ that grants measure $\mu_{\varrho^\star}(v)$ to $v$. In principle, more than one such move may exist and any one of them can serve as witness.

The solution corresponds to the inflationary fixpoint [10, 33] of the two phases mentioned above, $\mathsf{sol} \triangleq \mathsf{ifp}\, \varrho \,.\, \mathsf{prg}_+(\mathsf{prg}_0(\varrho)) \colon \mathrm{QDR} \rightharpoonup \mathrm{QDR}$, defined by the progress operators $\mathsf{prg}_0$ and $\mathsf{prg}_+$. The first phase is computed by the operator $\mathsf{prg}_0 \colon \mathrm{QDR} \rightharpoonup \mathrm{QDR}$ as follows: $\mathsf{prg}_0(\varrho) \triangleq \mathsf{sup}\{\varrho, \mathsf{lift}(\varrho, \overline{\mathsf{Q}(\varrho)}, \mathrm{Ps})\}$. This operator is responsible of enforcing the progress condition on the positions outside the quasi dominion $\mathsf{Q}(\varrho)$ that do not satisfy the inequalities between the measures along a move leading to $\mathsf{Q}(\varrho)$ itself. It does that by applying the lift operator with $\overline{\mathsf{Q}(\varrho)}$ as source and no restrictions on the moves. Those position that acquire a positive measure in this phase contribute to enlarging the current quasi dominion. Observe that the strategy component of the QDR is updated so that it is a $\oplus$-witness of the new quasi dominion. To guarantee that measures never decrease, the supremum *w.r.t.* the QDR-space ordering is taken as result.

**Lemma 1.** *$\mu_\varrho$ is a progress measure over $\overline{\mathsf{Q}(\varrho)}$, for all fixpoints $\varrho$ of* $\mathsf{prg}_0$.

The second phase, instead, implements the mechanism intuitively described above, while analyzing the simple example of Figure 1. This is achieved by the operator $\mathsf{prg}_+$ reported in Algorithm 1. The procedure iteratively examines the current quasi dominion and lifts the measures of the positions that must exit from it. Specifically, it processes $\mathsf{Q}(\varrho)$ layer by layer, starting from the outer layer of positions that must escape. The process ends when a, possibly empty, closed weak quasi dominion is obtained. Recall that all the positions in a closed weak quasi dominion are necessarily winning for player $\oplus$, due to Proposition 1. We distinguish two sets of positions in $\mathsf{Q}(\varrho)$. Those that already satisfy the progress condition and those that do not. The measures of first ones already witness an escape route from $\mathsf{Q}(\varrho)$. The other ones, instead, are those whose current choice is to remain inside it. For instance, when considering the measure function $\mu_2$ in the example of Figure 1, position $\mathsf{a}$ belongs to the first set, while positions $\mathsf{c}$ and $\mathsf{d}$ to the second one, since the choice of $\mathsf{c}$ is to follow the internal move $(\mathsf{c}, \mathsf{d})$.

Since the only positions that change measure are those in the second set, only such positions need to be examined. To identify them, which form a weak quasi dominion $\mathsf{\Delta}(\varrho)$ strictly contained in $\mathsf{Q}(\varrho)$, we proceed as follows. First, we collect the set $\mathsf{npp}(\varrho)$ of positions in $\mathsf{Q}(\varrho)$ that do not satisfy the progress condition, called the *non-progress positions*. Then, we compute the set of positions that will have no choice other than reaching $\mathsf{npp}(\varrho)$, by computing the inflationary fixpoint of a suitable $\mathsf{pre}$ operator.

$$\begin{aligned}\mathsf{npp}(\varrho) &\triangleq \{v \in \mathsf{Q}(\varrho) \cap \mathrm{Ps}_{\oplus} : \exists u \in Mv(v) \,.\, \mu_{\varrho}(v) < \mu_{\varrho}(u) + v\} \\ &\cup \{v \in \mathsf{Q}(\varrho) \cap \mathrm{Ps}_{\boxminus} : \forall u \in Mv(v) \,.\, \mu_{\varrho}(v) < \mu_{\varrho}(u) + v\}. \\ \mathsf{pre}(\varrho, \mathrm{Q}) &\triangleq \mathrm{Q} \cup \{v \in \mathsf{Q}(\varrho) \cap \mathrm{Ps}_{\oplus} : \sigma_{\varrho}(v) \in \mathrm{Q}\} \\ &\cup \{v \in \mathsf{Q}(\varrho) \cap \mathrm{Ps}_{\boxminus} : \forall u \in Mv(v) \setminus \mathrm{Q} \,.\, \mu_{\varrho}(v) < \mu_{\varrho}(u) + v\}.\end{aligned}$$

The final result is $\mathsf{\Delta}(\varrho) \triangleq (\mathsf{ifp}\,\mathrm{Q}\,.\,\mathsf{pre}(\varrho, \mathrm{Q}))(\mathsf{npp}(\varrho))$. Intuitively, $\mathsf{\Delta}(\varrho)$ contains all the $\oplus$-positions that are forced to reach $\mathsf{npp}(\varrho)$ via the quasi-dominion $\oplus$-witness and all the $\boxminus$-positions that can only avoid reaching $\mathsf{npp}(\varrho)$ by strictly increasing their measure, which player $\boxminus$ wants obviously to prevent.

It is important to observe that, from a functional view-point, the progress operator $\mathsf{prg}_{+}$ would work just as well if applied to the entire quasi dominion $\mathsf{Q}(\varrho)$, since it would simply leave unchanged the measure of those positions that already satisfy the progress condition. However, it is crucial that only the positions in $\mathsf{\Delta}(\varrho)$ are processed in order to achieve the best asymptotic complexity bound known to date. We shall reiterate on this point later on.

At each iteration of the while-loop of Algorithm 1, let Q denote the current (weak) quasi dominion, initially set to $\mathsf{\Delta}(\varrho)$ (Line 1). It first identifies the positions in Q that can immediately escape from it (Line 2). Those are *(i)* all the $\boxminus$-position with a move leading outside of Q and *(ii)* the $\oplus$-positions $v$ whose $\oplus$-witness $\sigma_{\varrho}$ forces $v$ to exit from Q, namely $\sigma_{\varrho}(v) \notin \mathrm{Q}$, and that cannot strictly increase their measure by choosing to remain in Q. While the condition for $\boxminus$-position is obvious, the one for $\oplus$-positions require some explanation. The crucial observation here is that, while player $\oplus$ does indeed prefer to remain in the quasi dominion, it can only do so while ensuring that by changing strategy it does not enable infinite plays within Q that are winning for the adversary. In other words, the new $\oplus$-strategy must still be a $\oplus$-witness for Q and this can only be ensured if the new choice strictly increases its measure. The operator $\mathsf{esc}\colon \mathrm{QDR} \times 2^{\mathrm{Ps}} \to 2^{\mathrm{Ps}}$ formalizes the idea:

**Alg. 1:** Progress Operator

**signature** $\mathsf{prg}_{+} : \mathrm{QDR} \rightharpoonup \mathrm{QDR}$
**function** $\mathsf{prg}_{+}(\varrho)$
1 $\quad \mathrm{Q} \leftarrow \mathsf{\Delta}(\varrho)$
2 $\quad$ **while** $\mathsf{esc}(\varrho, \mathrm{Q}) \neq \emptyset$ **do**
3 $\quad\quad \mathrm{E} \leftarrow \mathsf{bep}(\varrho, \mathrm{Q})$
4 $\quad\quad \varrho \leftarrow \mathsf{lift}(\varrho, \mathrm{E}, \overline{\mathrm{Q}})$
5 $\quad\quad \mathrm{Q} \leftarrow \mathrm{Q} \setminus \mathrm{E}$
6 $\quad \varrho \leftarrow \mathsf{win}(\varrho, \mathrm{Q})$
7 $\quad$ **return** $\varrho$

$$\begin{aligned}\mathsf{esc}(\varrho, \mathrm{Q}) &\triangleq \{v \in \mathrm{Q} \cap \mathrm{Ps}_{\boxminus} : Mv(v) \setminus \mathrm{Q} \neq \emptyset\} \\ &\cup \{v \in \mathrm{Q} \cap \mathrm{Ps}_{\oplus} : \sigma_{\varrho}(v) \notin \mathrm{Q} \wedge \forall u \in Mv(v) \cap \mathrm{Q} \,.\, \mu_{\varrho}(u) + v \leq \mu_{\varrho}(v))\}.\end{aligned}$$

Consider, for instance, the example in Figure 2 and a QDR $\varrho$ such that $\mu_{\varrho} = \{\mathtt{a} \mapsto 3; \mathtt{b} \mapsto 2; \mathtt{c}, \mathtt{d}, \mathtt{f} \mapsto 1; \mathtt{e} \mapsto 0\}$ and $\sigma_{\varrho} = \{\mathtt{b} \mapsto \mathtt{a}; \mathtt{f} \mapsto \mathtt{d}\}$. In this case, we have $\mathrm{Q}_{\varrho} = \{\mathtt{a}, \mathtt{b}, \mathtt{c}, \mathtt{d}, \mathtt{f}\}$ and $\Delta(\varrho) = \{\mathtt{c}, \mathtt{d}, \mathtt{f}\}$, since c is the only non-progress positions, d is forced to follow c in order to avoid the measure increase required to reach b, and f is forced by the $\oplus$-witness to reach d. Now, consider the situation where the current weak quasi dominion is $\mathrm{Q} = \{\mathtt{c}, \mathtt{f}\}$, *i.e.* after d has escaped from $\Delta(\varrho)$. The escape set of Q is $\{\mathtt{c}, \mathtt{f}\}$. To see why the $\oplus$-position f is escaping, observe that $\mu_{\varrho}(\mathtt{f}) + \mathtt{f} = 1 = \mu_{\varrho}(\mathtt{f})$ and that, indeed, should player $\oplus$ choose to change its strategy and take the move $(\mathtt{f}, \mathtt{f})$ to remain in Q, it would obtain an infinite play with payoff 0, thus violating the definition of weak quasi dominion.

Before proceeding, we want to stress an easy consequence of the definition of the notion of escape set and Conditions 1c and 1d of Definition 4, *i.e.*, that every escape position of the quasi dominion $\mathsf{Q}(\varrho)$ can only assume its weight as possible measure inside a QDR $\varrho$, as reported is the following proposition. This observation, together with Proposition 2, ensures that the measure of a position $v \in \mathsf{Q}(\varrho)$ is an under approximation of the weight of all finite plays leaving $\mathsf{Q}(\varrho)$.

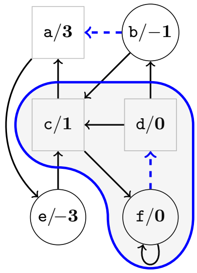

Fig. 2: Another MPG.

**Proposition 4.** *Let $\varrho$ be a* QDR*. Then, $\mu_{\varrho}(v) = \mathsf{wg}(v) > 0$, for all $v \in \mathsf{esc}(\varrho, \mathsf{Q}(\varrho))$.*

Now, going back to the analysis of the algorithm, if the escape set is non-empty, we need to select the escape positions that need to be lifted in order to satisfy the progress condition. The main difficulty is to do so in such a way that the resulting measure function still satisfies Condition 1d of Definition 4, for all the $\boxminus$-positions with positive measure. The problem occurs when a $\boxminus$-position can exit either immediately or passing through a path leading to another position in the escape set. Consider again the example above, where $\mathrm{Q} = \Delta(\varrho) = \{\mathtt{c}, \mathtt{d}, \mathtt{f}\}$. If position d immediately escapes from Q using the move $(\mathtt{d}, \mathtt{b})$, it would change its measure to $\mu'(\mathtt{d}) = \mu(\mathtt{b}) + \mathtt{d} = 2 > \mu(\mathtt{d}) = 1$. Now, position c has two ways to escape, either directly with move $(\mathtt{c}, \mathtt{a})$ or by reaching the other escape position d passing through f. The first choice would set its measure to $\mu(\mathtt{a}) + \mathtt{c} = 4$. The resulting measure function, however, would not satisfy Condition 1d of Definition 4, as the new measure of c would be greater than $\mu'(\mathtt{d}) + \mathtt{c} = 2$, preventing to obtain a QDR. Similarly, if position d escapes from Q passing through c via the move $(\mathtt{c}, \mathtt{a})$, we would have $\mu''(\mathtt{d}) = \mu''(\mathtt{c}) + \mathtt{d} = (\mu(\mathtt{a}) + \mathtt{c}) + \mathtt{d} = 4 > 2 = \mu(\mathtt{b}) + \mathtt{d}$, still violating Condition 1d. Therefore, in this specific case, the only possible way to escape is to reach b. The solution to this problem is simply to lift in the current iteration only those positions that obtain the lowest possible measure increase, hence position d in the example, leaving the lift of c to some subsequent iteration of the algorithm that would choose the correct escape route via d. To do so, we first compute the minimal measure increase, called the *best-escape forfeit*, that each position in the escape set would obtain by exiting the quasi dominion immediately. The positions with the lowest possible forfeit, called *best-escape positions*, can all be lifted at the same time. The intuition is that the measure of all the positions that escape from a (weak) quasi dominion will necessarily be increased of at least the minimal best-escape forfeit. This observation is at the core of the proof of Theorem 2

(see the appendix) ensuring that the desired properties of QDRs are preserved by the operator $\mathsf{prg}_{+}$. The set of best-escape positions is computed by the operator $\mathsf{bep}\colon \mathrm{QDR}\times 2^{\mathrm{Ps}} \to 2^{\mathrm{Ps}}$ as follows: $\mathsf{bep}(\varrho, \mathrm{Q}) \triangleq \mathsf{argmin}_{v\in\mathsf{esc}(\varrho,\mathrm{Q})} \mathsf{bef}(\mu_{\varrho}, \mathrm{Q}, v)$, where the operator $\mathsf{bef}\colon \mathrm{MF}\times 2^{\mathrm{Ps}}\times \mathrm{Ps} \to \mathbb{N}_{\infty}$ computes, for each position $v$ in a quasi dominion Q, its best-escape forfeit:

$$\mathsf{bef}(\mu, \mathrm{Q}, v) \triangleq \begin{cases} \mathsf{max}\{\mu(u) + v - \mu(v) : u \in Mv(v) \setminus \mathrm{Q}\}, & \text{if } v \in \mathrm{Ps}_{\oplus}; \\ \mathsf{min}\{\mu(u) + v - \mu(v) : u \in Mv(v) \setminus \mathrm{Q}\}, & \text{otherwise.} \end{cases}$$

In our example, $\mathsf{bef}(\mu, \mathrm{Q}, \mathsf{c}) = \mu(\mathsf{a}) + \mathsf{c} - \mu(\mathsf{c}) = 4 - 1 = 3$, while $\mathsf{bef}(\mu, \mathrm{Q}, \mathsf{d}) = \mu(\mathsf{b}) + \mathsf{d} - \mu(\mathsf{d}) = 2 - 1 = 1$. Therefore, $\mathsf{bep}(\varrho, \mathrm{Q}) = \{\mathsf{d}\}$.

Once the set E of best-escape positions is identified (Line 3), the procedure lifts them restricting the possible moves to those leading outside the current quasi dominion (Line 4). Those positions are, then, removed from the set (Line 5), thus obtaining a smaller weak quasi dominion ready for the next iteration.

The algorithm terminates when the (possibly empty) current quasi dominion Q is closed. By virtue of Proposition 1, all those positions belong to $\mathrm{Wn}_{\oplus}$ and their measure is set to $\infty$ by means of the operator $\mathsf{win}\colon \mathrm{QDR}\times 2^{\mathrm{Ps}} \rightharpoonup \mathrm{QDR}$ (Line 6), which also computes the winning $\oplus$-strategy on those positions, as follows: $\mathsf{win}(\varrho, \mathrm{Q}) \triangleq \varrho^{\star}$, where $\mu_{\varrho^{\star}} \triangleq \mu_{\varrho}[\mathrm{Q} \mapsto \infty]$ and, for all $\oplus$-positions $v \in \mathsf{Q}(\varrho^{\star}) \cap \mathrm{Ps}_{\oplus}$, we choose $\sigma_{\varrho^{\star}}(v) \in \mathsf{argmax}_{u\in Mv(v)\cap \mathrm{Q}}\ \mu_{\varrho}(u) + v$, if $\sigma_{\varrho}(v) \not\in \mathrm{Q}$, and $\sigma_{\varrho^{\star}}(v) = \sigma_{\varrho}(v)$, otherwise. Observe that, since we know that every $\oplus$-position $v \in \mathrm{Q} \cap \mathrm{Ps}_{\oplus}$, whose current $\oplus$-witness leads outside Q, is not an escape position, any move $(v, u)$ within Q that grants the maximal stretch $\mu_{\varrho}(u) + v$ strictly increases its measure and, therefore, is a possible choice for a $\oplus$-witness of the $\oplus$-dominion Q.

At this point, it should be quite evident that the progress operator $\mathsf{prg}_{+}$ is responsible of enforcing the progress condition on the positions inside the quasi dominion $\mathsf{Q}(\varrho)$, thus, the following necessarily holds.

**Lemma 2.** *$\mu_{\varrho}$ is a progress measure over $\mathsf{Q}(\varrho)$, for all fixpoints $\varrho$ of $\mathsf{prg}_{+}$.*

In order to prove the correctness of the proposed algorithm, we first need to ensure that any quasi-dominion space $\mathcal{Q}$ is indeed closed under the operators $\mathsf{prg}_{0}$ and $\mathsf{prg}_{+}$. This is established by the following theorem, which states that the operators are total functions on that space.

**Theorem 2.** *The operators $\mathsf{prg}_{0}$ and $\mathsf{prg}_{+}$ are total inflationary functions.*

Since both operators are inflationary, so is their composition, which admits fixpoint. Therefore, the operator $\mathsf{sol}$ is well defined. Moreover, following the same considerations discussed at the end of Section 3, it can be proved the fixpoint is obtained after at most $n \cdot (S + 1)$ iterations. Let $\mathsf{ifp}_k\, X \,.\, \mathsf{F}(X)$ denote the $k$-th iteration of an inflationary operator $\mathsf{F}$. Then, we have the following theorem.

**Theorem 3 (Termination).** *The solver operator $\mathsf{sol} \triangleq \mathsf{ifp}\, \varrho \,.\, \mathsf{prg}_{+}(\mathsf{prg}_{0}(\varrho))$ is a well-defined total function. Moreover, for every $\varrho \in \mathrm{QDR}$ it holds that $\mathsf{sol}(\varrho) = (\mathsf{ifp}_k\, \varrho^{\star} \,.\, \mathsf{prg}_{+}(\mathsf{prg}_{0}(\varrho^{\star})))(\varrho)$, for some index $k \leq n\cdot(S+1)$, where $n$ is the number of positions in the MPG and $S \triangleq \sum\{\mathsf{wg}(v) \in \mathbb{N} : v \in \mathrm{Ps} \wedge \mathsf{wg}(v) > 0\}$ the total sum of its positive weights.*

As already observed before, Figure 1 exemplifies an infinite family of games with a fixed number of positions and increasing maximal weight $k$ over which the SEPM algorithm requires $2k+1$ iterations of the lift operator. On the contrary, QDPM needs exactly two iterations of the solver operator $\mathsf{sol}$ to find the progress measure, starting from the smallest measure function $\mu_0$. Indeed, the first iteration returns a measure function $\mu_1 = \mathsf{sol}(\mu_0)$, with $\mu_1(\mathtt{a}) = k$, $\mu_1(\mathtt{b}) = \mu_1(\mathtt{c}) = 0$, and $\mu_1(\mathtt{d}) = 1$, while the second one $\mu_2 = \mathsf{sol}(\mu_1)$ identifies the smallest progress measure, with $\mu_1(\mathtt{a}) = \mu_1(\mathtt{c}) = k$, $\mu_1(\mathtt{b}) = 0$, and $\mu_1(\mathtt{d}) = k+1$. From this observations, the next result immediately follows.

**Theorem 4.** *An infinite family of* MPG*s* $\{\eth_k\}_k$ *exists on which* QDPM *requires a constant number of measure updates, while* SEPM *requires* $\mathrm{O}(k)$ *such updates.*

From Theorem 1 and Lemmas 1 and 2 it follows that the solution provided by the algorithm is indeed a progress measure, hence establishing soundness. Completeness follows from Theorem 3 and from Condition 1b of Definition 4 that ensures that all the positions with infinite measure are winning for player $\oplus$.

**Theorem 5 (Correctness).** $\|\mathsf{sol}(\varrho)\|_{\boxminus} = \mathrm{Wn}_{\boxminus}$*, for every* $\varrho \in \mathrm{QDR}$.

The following lemma ensures that each execution of the operator $\mathsf{prg}_+$ strictly increases the measure of all the positions in $\Delta(\varrho)$.

**Lemma 3.** *Let* $\varrho^\star \triangleq \mathsf{prg}_+(\varrho)$*. Then,* $\mu_{\varrho^\star}(v) > \mu_\varrho(v)$*, for all positions* $v \in \Delta(\varrho)$.

Recall that each position can at most be lifted $S+1 = \mathrm{O}(n \cdot W)$ times and, by the previous lemma, the complexity of $\mathsf{sol}$ only depends on the cumulative cost of such lift operations. We can express, then, the total cost as the sum, over the set of positions in the game, of the cost of all the lift operations performed on that positions. Each such operation can be computed in time linear in the number of incoming and outgoing moves of the corresponding lifted position $v$, namely $\mathrm{O}\big((|Mv(v)| + |Mv^{-1}(v)|) \cdot \log S\big)$, with $\mathrm{O}(\log S)$ the cost of each arithmetic operation involved. Summing all up, the actual asymptotic complexity of the procedure can, therefore, be expressed as $\mathrm{O}(n \cdot m \cdot W \cdot \log(n \cdot W))$.

**Theorem 6 (Complexity).** QDPM *requires time* $\mathrm{O}(n \cdot m \cdot W \cdot \log(n \cdot W))$ *to solve an* MPG *with $n$ positions, $m$ moves, and maximal positive weight $W$.*

### 5 Experimental Evaluation

In order to assess the effectiveness of the proposed approach, we implemented both QDPM and SEPM [13], the most efficient known solution to the problem and the more closely related one to QDPM, in C++ within Oink [32]. Oink has been developed as a framework to compare parity game solvers. However, extending the framework to deal with MPGs is not difficult. The form of the arenas of the two types of games essentially coincide, the only relevant difference being that MPGs allow negative numbers to label game positions. We ran the two solvers against randomly generated MPGs of various sizes. [1]

[1] The experiments were carried out on a 64-bit 3.9GHz quad-core machine, with Intel i5-6600K processor and 8GB of RAM, running Ubuntu 18.04.

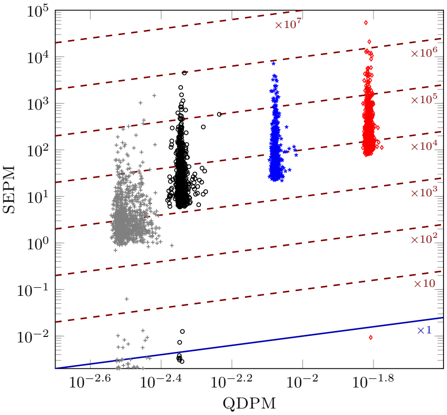

Fig. 3: Random games with $10^4$ positions.

Figure 3 compares the solution time, expressed in seconds, of the two algorithms on 4000 games, each with $10^4$ positions and randomly assigned weights in the range $[-15 \times 10^3, 15 \times 10^3]$. The scale of both axes is logarithmic. The experiments are divided in 4 clusters, each containing 1000 games. The benchmarks in different clusters differ in the maximal number $m$ of outgoing moves per position, with $m \in \{10, 20, 40, 80\}$. These experiments clearly show that QDPM substantially outperforms SEPM. Most often, the gap between the two algorithms is between two and three orders of magnitude, as indicated by the dashed diagonal lines. It also shows that SEPM is particularly sensitive to the density of the underlying graph, as its performance degrades significantly as the number of moves increases. The maximal solution time was 21000 sec. for SEPM and 0.017 sec. for QDPM. Figure 4, instead, compares the two algorithms fixing the maximal out-degree of the underlying graphs to 2, in the left-hand picture, and to 40, in the right-hand one, while increasing the number of positions from $10^3$ to $10^5$ along the x-axis. Each picture displays the performance results on 2800 games. Each point shows the total time to solve 100 randomly generated games with that given number of positions, which increases by 1000 up to size $2 \cdot 10^3$ and by 10000, thereafter. In both pictures the scale is logarithmic. For the experiments in the right-hand picture we had to set a timeout for SEPM to 45 minutes per game, which was hit most of the times on the bigger ones. Once again, the QDPM significantly outperforms SEPM on both kinds of benchmarks, with a gap of more than an order of magnitude on the first ones, and a gap of more than three orders of magnitude on the second ones. The results also confirm that the performance gap grows considerably as the number of moves per position increases.

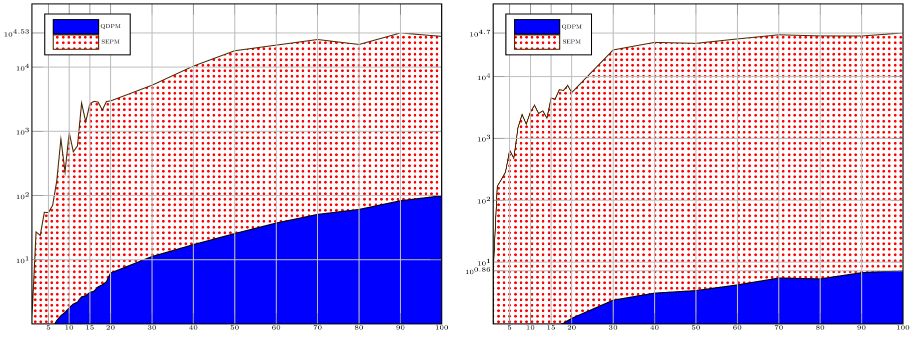

Fig. 4: Total solution times in seconds of SEPM and QDPM on 5600 random games.

We are not aware of actual concrete benchmarks for MPGs. However, exploiting the standard encoding of parity games into mean-payoff games [25], we can compare the behavior of SEPM and QDPM on concrete verification problems encoded as parity games. For completeness, Table 1 reports some experiments on such problems. The table reports the execution times, expressed in seconds, required by the two algorithms to solve instances of two classic verification problems: the Elevator Verification and the Language Inclusion problems. These two benchmarks are included in the PGSolver [23] toolkit and are often used as benchmarks for parity games solvers. The first benchmark is a *verification under fairness* constraints of a simple model of an elevator, while the second one encodes the *language inclusion* problem between a non-deterministic Büchi automaton and a deterministic one. The results on various instances of those problems confirm that QDPM significantly outperforms the classic progress measure approach. Note also that the translation into MPGs, which encodes priorities as weights whose absolute value is exponential in the values of the priorities, leads to games with weights of high magnitude. Hence, the results in Table 1 provide further evidence that QDPM is far less dependent on the absolute value of the weights. They also show that QDPM can be very effective for the solution of real-world qualitative verification problems. It is worth noting, though, that the translation from parity to MPGs gives rise to weights that are exponentially distant from each other [25]. As a consequence, the resulting benchmarks are not necessarily representative of MPGs, being a very restricted subclass. Nonetheless, they provide evidence of the applicability of the approach in practical scenarios.

| Benchmark | Positions | Moves | SEPM | QDPM |
|---|---|---|---|---|
| Elevator 1 | 144 | 234 | 0.0661 | **0.0001** |
| Elevator 2 | 564 | 950 | 8.80 | **0.0003** |
| Elevator 3 | 2688 | 4544 | 4675.71 | **0.0017** |
| Lang. Incl. 1 | 170 | 1094 | 3.18 | **0.0021** |
| Lang. Incl. 2 | 304 | 1222 | 16.76 | **0.0019** |
| Lang. Incl. 3 | 428 | 878 | 20.25 | **0.0033** |
| Lang. Incl. 4 | 628 | 1538 | 135.51 | **0.0029** |
| Lang. Incl. 5 | 509 | 2126 | 148.37 | **0.0034** |
| Lang. Incl. 6 | 835 | 2914 | 834.90 | **0.0051** |
| Lang. Incl. 7 | 1658 | 4544 | 2277.87 | **0.0100** |

Table 1: Concrete verification problems.

## 6 Concluding Remarks

We proposed a novel solution algorithm for the decision problem of MPGs that integrates progress measures and quasi dominions. We argue that the integration of these two concepts may offer significant speed up in convergence to the solution, at no additional computational cost. This is evidenced by the existence of a family of games on which the combined approach can perform arbitrarily better than a classic progress measure based solution. Experimental results also show that the introduction of quasi dominions can often reduce solution times up to three order of magnitude, suggesting that the approach may be very effective in practical applications as well. We believe that the integration approach we devised is general enough to be applied to other types of games. In particular, the application of quasi dominions in conjunction with progress measure based approaches, such as those of [27] and [21], may lead to practically efficient quasi polynomial algorithms for parity games and their quantitative extensions.
